# Perceived Neighborhood and Home Environmental Factors Associated with Television Viewing among Taiwanese Older Adults

**DOI:** 10.3390/ijerph13070708

**Published:** 2016-07-13

**Authors:** Ming-Chun Hsueh, Yung Liao, Shao-Hsi Chang

**Affiliations:** 1 Department of Physical Education, National Taiwan Normal University, 162, Heping East Road Section 1, Taipei 106, Taiwan; boxeo19912016@gmail.com; 2 Department of Health Promotion and Health Education, National Taiwan Normal University, Taipei 106, Taiwan; liaoyung@ntnu.edu.tw

**Keywords:** sedentary behavior, built environment, traffic safety, household, sedentary equipment, elderly, Asia

## Abstract

This study examined the associations between perceived neighborhood and home environmental factors and excessive television (TV) viewing time among Taiwanese older adults. The sample data was collected by administering computer-assisted telephone interviewers to 980 Taiwanese older adults (aged ≥ 65 years) living in two regions. Odds ratios (ORs) and 95% confidence intervals (CIs) were calculated to examine the associations between self-reported perceived neighborhood and home environmental attributions and TV viewing time by using logistic regression analyses. The results showed that perceived neighborhood and home environmental factors were associated with excessive TV viewing time (≥2 h/day) after adjusting for potential confounders. Compared with a reference group, older adults who perceived their neighborhoods to have unsafe traffic were more likely to report excessive TV viewing time (OR = 1.36, 95%CI = 1.02–1.82). Older adults who reported having two or more TV sets in the home (OR = 1.77, CI = 1.28–2.44) and having a TV in the bedroom (OR = 1.55, CI = 1.18–2.03) were also more likely to report excessive TV viewing time. Further longitudinal research can confirm these findings, and tailored interventions focusing on the perceptions of neighborhood traffic safety and TV access at home for older adults might be effective means of preventing excessive TV viewing time.

## 1. Introduction

Sedentary behavior has become a new focus of public health researchers and officials, as recent research has indicated that excessive amounts of sedentary behavior may negatively affect peoples’ health over time [1], with older adults also likely being affected [2]. Television (TV) viewing is one commonly engaged form of sedentary behavior [3] that occupies a large proportion of the leisure time of older adults [4]. Several prospective studies have reported that prolonged TV viewing time is associated with increased risk of metabolic syndrome, increased waist circumference, obesity and cardiovascular disease [5,6].

Nonetheless, on a global basis, almost 56% of older adults report watching more than 2 h of TV per day [4]. Relatedly, conclusive evidence has indicated that 2 h of TV viewing per day serves a reasonable cutoff point, with more than 2 h per day having been clearly associated with deleterious health outcomes [7]. Several previous studies have shown viewing more than 2 h of TV per day is associated with increased risk of overweight [8,9] and type 2 diabetes [10]. Despite these adverse consequences, a recent study found that nearly half (47.4%) of older adults in Taiwan engage in excessive TV viewing time (≥2 h/day) [11]. Considering the rapidly aging population of Taiwan and the high prevalence of TV viewing behavior in this older age group, the aforementioned findings regarding the health risks of excessive sedentary behavior imply that it is critical for policy makers and intervention designers to develop effective strategies to prevent excessive TV viewing time among Taiwanese older adults.

In terms of utilizing an ecological approach in designing effective interventions and relevant policies, it is important to understand how environmental attributes correlate with sedentary behavior [12]. Yen [13] proposed that immediate neighborhood environmental attributes, such as traffic safety, crime levels and access to or the quality of commercial and public services, might be relevant to older adults’ health-related behaviors. For example, a longitudinal study conducted in Australia found that older adults who did not perceive local traffic to be a deterrent to spending time outside the home engaged in less TV viewing time than those who perceived traffic to be a deterrent [14]. Another cross-sectional study of older adults in Belgium found that those who reported positive perceptions of their neighborhoods in terms of access to facilities, safety from crime and walking facilities viewed less TV [15]. These studies suggest that broader contextual factors and perceived neighborhood environments may play crucial roles in older adults’ sedentary behavior, particularly TV viewing. However, despite the fact that the relationship between environmental attributes and TV viewing behavior might vary according to culture and environment [16], but limitation of studies to date have examined these relationships on Asia’s older adults, particularly in Taiwan.

Although studies on the relationships between perceived neighborhood environments and older adults’ TV viewing behaviors are just beginning to emerge, research regarding the relationships between home environments and TV viewing levels among older adults continues to be lacking. Owen et al. [12] indicated that aspects of the home environment may also potentially influence TV viewing. In particularly, for TV viewing behavior, previous research has focused specifically on the access to a TV within the home, which is a crucial home environmental factor and includes TV placement, such as a TV in the bedroom, and the number of TV sets in home; these factors have been found to be related to excessive TV viewing [17,18,19]. However, previous findings concerning the associations between home environments and TV viewing time were mostly in regards to children [17], youth [18] and adult populations [19]; few studies have focused on older adults specifically. Moreover, the previously reported findings regarding associations between the home environment and TV viewing time are inconsistent [17,18,19]. However, since the vast majority of TV viewing time can be expected to be accumulated within the home environment, there are relatively few empirical studies that have investigated possible correlations between specific aspects of the home environment and TV viewing time among older adults [15]. To our knowledge, no study has yet concurrently explored perceived neighborhood and home environmental factors for associations with TV viewing time in older adults, in spite of the fact that such investigations could potentially provide more practical and policy-related information. Therefore, the main aim of this study was to examine the associations between perceived neighborhood and home environmental factors with TV viewing time among Taiwanese older adults.

## 2. Methods

### 2.1. Participants

The target population of this study consisted of 468,922 older adults aged over 65 years living in Taipei City and Chiayi County. Taipei City covers 271.8 km^2^ and has a population of 380,527 older adults, while Chiayi County covers 1903.6 km^2^ and has a population of 88,395 older adults. The two areas were selected because of their high aging index relative to the rest of Taiwan. Taipei City (14.08%) and Chiayi County (16.84%) had the highest percentages of older adults in their overall populations among Taiwan’s metropolitan and nonmetropolitan areas, respectively.

A cross-sectional survey was conducted using a computer-assisted telephone interview (CATI) system in May 2015. The required sample size for this study was calculated to be 1068 older adults with a 95% confidence level and a 3% confidence interval. A stratified random sampling proportionate based on gender (women and men) and the two municipalities (Taipei City and Chiayi County) was used. Sampling was performed through a random-digit-dialing telephone-based survey from a national database. This database covers all residential telephone numbers, with each residential telephone number having an equal probability of being called. A total of 1714 older adults were asked to participate, and 1095 of them completed the survey (response rate: 63.9%). No incentive was offered to the respondents. This study protocol was approved by the Ethics Committee of National Taiwan Normal University (201504HM004).

### 2.2. Measures

#### 2.2.1. Outcome Variables

The participants reported their total time spent watching TV or video/DVD in hours and minutes during the past week. This item has been shown to have good test–retest reliability (intraclass correlation coefficient (ICC) = 0.76; Spearman ρ = 0.78) in the Measure of Older Adults’ Sedentary Time questionnaire (MOST) [20]. The MOST questionnaires were translated into Chinese according to the World Health Organization (WHO) process of instrument translation and adaptation [21], and the translated questionnaire was then submitted to a seven-day test-retest reliability study in a sample of 53 older Taiwanese adults. The item regarding TV viewing time has been shown to have an acceptable level of test-retest reliability (ICC = 0.69; Spearman ρ = 0.67), as measured according to study criteria used by Gardiner et al. [20]. The sum of total TV viewing time was dichotomized based on the median value into two categories, namely, excessive TV viewing time (≥2 h/day) and low TV viewing time (<2 h/day). This cutoff point (≥2 h/day) was also reported as being associated with health risks in previous studies [7].

#### 2.2.2. Perceived Neighborhood Environmental Variables

The questionnaire utilized for the perceived neighborhood environmental variables was taken from the International Physical Activity Questionnaire-Environmental Module (IPAQ-E). A Taiwanese version of the IPAQ-E has previously been used to examine the relationships between levels of walking and cycling for transportation [22]. The IPAQ-E consists of 17 questions: seven core items, four recommended items, and six optional items. In this study, only select core and recommended items were used (Table 1).

Nine of 10 items, excluding residential density, these items refer to the neighborhood environment within a 10- to 15-min walk from the given respondent’s residence. The item of residential density was the five options were as follows: detached single-family housing; townhouses, row houses, apartments or condos of 2–3 stories; a mix of single-family residences and townhouses, row houses, apartments or condos; apartments or condos of 4–12 stories; and apartments or condos of more than 12 stories. The final item of “household motor vehicles” was categorized as a confounding variable and not included in Table 1. All of items were converted into binary items. For residential density, the choice of detached single-family residences formed a category indicating “low residential density”, while the other possible responses were included in another category indicating “high residential density”. With regard to the other questions, responses were classified into two categories of “agree” (strongly agree and somewhat agree) and “disagree” (somewhat disagree and strongly disagree). The item of “household motor vehicles” was divided into “one or more” and “none”, and was categorized as a confounding variable. These classifications were similar to those used in previous studies [22,23].

#### 2.2.3. Home Environmental Variables

The survey respondents were also asked two questions regarding home environmental factors affecting TV access, both of which were derived from a previously validated home electronic equipment scale [24]. Home environmental factors were measured with two items. The first was (1) Numbers of TV sets in home (*How many TV sets are in your household?*). This item was dichotomized based on the median value into two categories: “0–1” and “2 or more”. For this item, only 5 participants reported no TV at home; therefore, responses were combined into a “0–1” category. This category is in accordance with the previous studies for the home environment and TV viewing [25]. The second item was (2) TV set in bedroom (*Do you have a TV set in your bedroom?*). Both of these items have previously been used to measure the home environments of children, youth and adult populations [22,25,26].

#### 2.2.4. Potential Confounding Variables

The following potential confounding variables were assessed using an interviewer-administered questionnaire: age, gender, education level, marital status, living status, job status, residential area, body mass index (BMI), leisure-time physical activity (LTPA), and household motor vehicles. Age was divided into two categories: “65 to 74 years” and “≥75 years”. Education level was categorized into “college degree or more” and “up to high school”. Marital status was categorized into “married or unmarried”. Living status was divided into “living with others” and “living alone”. Job status was categorized into “employment” and “non-employment” (including retirement and unemployment). Residential area was divided into “metropolitan” and “nonmetropolitan”. BMI status was calculated from self-reported height (in meters) and weight (in kilograms) by dividing the weight by the height squared, and was classified into two categories determined by the Health Promotion Administration, Ministry of Health and Welfare, Taiwan: “non-overweight” (<24 kg/m^2^) and “overweight” (≥24 kg/m^2^) [27]. Another confounders was time spent in leisure-time physical activity (LTPA). LTPA was measured using the Taiwan version of the International Physical Activity Questionnaire, long version (IPAQ-LV) [28]. Participants were asked to recall the frequency and average duration of vigorous intensity leisure-time activity, moderate intensity leisure-time activity and walking during the last seven days. The questions included “During the last 7 days, on how many days did you do the activities (vigorous/moderate/walking) in your leisure time?” and “How much time did you usually spend on one of those days doing the activities in your leisure time?” The total amount of LTPA was classified into two groups: “sufficient LTPA” (≥150 min/week) and “insufficient LTPA” (<150 min/week). Sufficient LTPA refers to at least 150 min per week. This criterion is in accordance with the current recommendations for the practice of physical activity in older adult’s guidelines [29]. The number of household motor vehicles (none/one or more) was also regarded as a potential confounding variable. These factors have been associated with sedentary behaviors in previous studies [3,19,30,31].

### 2.3. Statistical Analyses

Following data cleaning, data from 980 Taiwanese older adults who fully completed the survey were included in the analysis. The Kolmogorov-Smirnov test revealed that the data regarding TV viewing time were not normally distributed (*p* < 0.001). Because the distribution of the TV viewing time data was skewed, binary logistic regression analyses were conducted to examine the relationships of each of the perceived neighborhood and home environmental variables separately with TV viewing time. Adjusted odds ratios (ORs) and 95% confidence intervals (CIs) were calculated for each variable. The level of significance was set at *p* < 0.05. All statistical analyses were performed with SPSS 24.0 for Windows (IBM, New York, NY, USA).

## 3. Results

### 3.1. Characteristics of Participants

Table 2 shows the personal characteristics of the study participants. The proportion of older adults aged over 75 years was 37.1%; 52.1% of the participants were men; 76.7% of the participants had been educated up to high school; 23.9% of the participants were unmarried; 13.1% of the participants were living alone; 80.4% of the participants were non-employed; 48.6% of the participants were living in a nonmetropolitan area; 41.4% of the participants were overweight; 40.7% of the participants reported insufficient LTPA; and 57.4% of the participants engaged in ≥2 h/day TV viewing time.

### 3.2. Descriptive Statistics for Perceived Neighborhood and Home Environmental Variables

Table 3 shows the descriptive statistics for the perceived neighborhood and home environmental variables. For perceived neighborhood environmental variables, 93.8% of older adults reported high residential density; 16.5% of the participants perceived poorly access to shops in neighborhood; 23.9% of the participants reported poorly access to public transport in neighborhood; 38% of the participants reported no presence of sidewalks in neighborhood; 41.6% of the participants reported no presence of bike lanes in neighborhood; 84.8% of the participants perceived their neighborhoods to be unsafe due to crime; 72.4% of the participants perceived their neighborhoods to be unsafe due to traffic; 23.5% of the participants perceived poor social environments in their neighborhood; 38.1% of the participants perceived poor aesthetics in their neighborhood; 81.2% of the participants owned cars or motor bikes. For home environmental variables, 23.5% of the participants reported two or more TV sets in their home. 39.5% of the participants owned a TV set in the bedroom.

Table 4 shows the adjusted logistic regression analysis results regarding the associations between perceived neighborhood and home environmental factors and excessive TV viewing time among older adults. With regard to the perceived neighborhood environmental factors, only older adults who reported unsafe traffic in their neighborhood were more likely to also report excessive time spent on TV viewing (OR, 1.36; 95%CI, 1.02–1.82) compared with those who perceived neighborhood traffic to be safe. There were no significant associations between any of the other perceived neighborhood environmental factors and excessive TV viewing time.

With regard to the home environmental factors, respondents who reported having ≥2 TV sets in the home were more likely to also report excessive TV viewing time (OR = 1.77, CI = 1.28–2.44) compared with those who reported having 0 or 1 TV set in the home. Moreover, respondents who reported having a TV set in the bedroom were more likely to also report excessive TV viewing time (OR = 1.55, CI = 1.18–2.03) compared with who reported having no TV set in the bedroom.

## 4. Discussion

The present study is the one of the few studies from an Asian country to have concurrently examined the associations of both perceived neighborhood and home environmental factors with TV viewing time among older adults. The most crucial findings of the present study are that, after controlling for the sociodemographic, BMI, LTPA and household motor vehicles covariates, older adults who perceived unsafe traffic, had two or more TV sets in the home and had a TV set in the bedroom were concurrently associated with excessive TV viewing time (≥2 h/day). Therefore, considering the influence of excessive TV viewing time on health risks, further cross-sectional and longitudinal research could be conducted to confirm these findings, while tailored interventions should focus on both perceived neighborhood and home environmental factors in order to encourage older adults to view less TV.

There are established relationships between neighborhood environmental factors and physical activity among older adults [32]. However, the relationship between the perceived environment and TV viewing time in older adults is not well understood, particularly in Asian countries. Our study revealed that Taiwanese older adults who perceived traffic in their neighborhood to be unsafe were also more likely to report excessive TV viewing time (≥2 h/day). This finding is consistent with those of previous studies in Western countries regarding adults in general [31] and Australian older adults in particular [14]. A possible explanation could be that various aspects of unsafe neighborhood traffic may make older adults afraid to go outside [33]. Such factors may include excessive noise, excessive vehicle speeds, inadequate signal times, and aggressive behavior on the part of other road users. This may cause them to spend less time walking for recreation in their neighborhood [34], which may, in turn, cause them to spend more time sitting at home while watching TV as an alternative to leisure time spent outside the home. In addition, while a previous study using nationally representative data showed that only 37.6% of Taiwanese adults perceived their neighborhood environments to have unsafe traffic [22], a higher percentage (72.4%) of the Taiwanese older adults living in Taipei City and Chiayi County surveyed in this study reported unsafe traffic in their neighborhoods. Therefore, it is possible that older adults may be more sensitive to “environmental threats” due to unsafe traffic in their neighborhood environments [14]. Worries about such threats could constrain older adults from engaging in some outdoor activities and make them more likely to watch TV at home as a leisure option. This result may strengthen the evidence for several perceived environmental factors associated with TV viewing, which is crucial for the literature because it thus far has a limited amount of data reported from Asia countries regarding older adults.

Furthermore, the present study found no significant associations between the other perceived environmental attributes examined (residential density, access to shops, access to public transport, presence of sidewalks, presence of bike lanes, access to recreational facilities, crime safety, social environment, aesthetics) and excessive TV viewing time. These results are consistent with previous studies on Australian older adults [14]. Shibata et al. found no associations between perceived environmental attributes (excluding unsafe traffic) and TV viewing time [14]. Some of these environmental characteristics may be favorably related to older adults’ walking, but do not appear to be related to their levels of TV viewing [14]. It is important to consider that TV viewing time and walking are independent behaviors that can have different determinants [35]. A possible explanation is that a larger neighborhood environment (e.g., residential density, presence of sidewalks and bike lanes, access to recreational facilities) is relevant for those who can walk a longer distance, but an environment in close vicinity to home may be more relevant to whether older adults spend more time at home [14]. Further studies exploring immediate environmental attributes close to home may be needed to identify environmental and policy initiatives to promote active living among older adults.

Regarding home environmental factors, previous studies have reported mixed results in terms of the relationships between the number of TV sets in the home and TV viewing times among different age levels [36]. For example, the number of TV sets in the home has been reported to be positively associated with TV viewing time in children [26], adolescents [37] and adults [38]. On the other hand, yet another study reported a negative association between that factor and TV viewing time in children [17], while still another reported no association between the factor and TV viewing in adults [19]. However, the present study found that older adults who reported having two or more TV sets in the home were more likely to also report excess TV viewing time (≥2 h/day). A possible reason is that greater access to TVs may be associated with more TV viewing time [38]. Moreover, an objective study that examined the context of sedentary behavior reported that older adults spend 70.1% of total sedentary time in the home and that an average of 84% of TV viewing time in screen time (include computer use) [39]. Therefore, in the home environment, reducing the number of TVs should be a priority when designing interventions to prevent excessive TV viewing time in older adults. In addition, consistent with previous studies in children and youth [17,18,24,26,40], we also found that having a TV in the bedroom was associated with excessive TV viewing time in older adults. A possible reason for this is that such aspects of the home environment could potentially influence an older adult’s health behaviors due to aging-related functional and mobility challenges [41,42], including challenges that would encourage sitting or lying on the bed and watching TV. Another possible season is that there is a strong social norm to locate TVs in the bedroom for the purpose of relaxing [12], even as many people may have limited knowledge of the potential detrimental health effects of having a TV in the bedroom [17]. Moreover, previously reported evidence has indicated that taking the TV out of the bedroom may help to decrease an adult’s TV viewing time [43]. Thus, older adults who have a TV set in the bedroom may spend more time at home and accumulate greater amounts of TV viewing time. Combined, these findings suggest that it may be important to target attributes of the home environments of older adults when implementing interventions to prevent prolonged sedentary behaviors.

The major strengths of this study were its large sample of Asian older adults recruited from diverse settings (i.e., metropolitan and nonmetropolitan areas) across Taiwan, as well as its examination of a broad range of perceived neighborhood and home environmental attributes. Several limitations need to be considered, however, in interpreting the present findings. First, the use of self-reported measures for the neighborhood attributes, home environmental attributes and TV viewing time could be subject to recall error and social desirability bias. Second, the study’s cross-sectional design limits the conclusions that can be drawn regarding the causality of the observed relationships between neighborhood and home environmental factors and TV viewing time. Third, the study participants did not constitute a nationally representative sample because they were limited to two localities and the study relied on a telephone-based survey. Fourth, other neighborhood factors, such as social environment [15] and neighborhood socio-economic status [13], as well as other home environmental factors, such as the size of the largest TV set [19] and the use of pay TV at home [25], were not measured, and could possibly have affected the TV viewing time results. Moreover, several studies have shown that demographics (e.g., gender and residential area) may have potential moderating effects between environmental attributes and TV viewing [25,40]; therefore, further study should consider examination of potential moderators. Furthermore, this study only measured TV viewing times; other domain-specific sedentary behaviors, such as computer use, reading, taking transportation and sedentary social activities, were not measured in this study [20]. Moreover, including segments of the population that did not have a household telephone (approximately 5.3% in 2013) was impossible [44].

## 5. Conclusions

This is the first study to concurrently examine the associations between both perceived neighborhood and home environmental factors and TV viewing time among older adults. Our findings contribute to the existing literature regarding excessive TV viewing time among older populations. Specifically, the main findings of the present study are that unsafe traffic, a high number of TV sets in the home and having a TV in the bedroom are associated with excessive TV viewing time among Taiwanese older adults. Further studies examining even more specific environmental attributes with objective behavioral measures are needed to better understand what environmental attributes should be specifically focused on in order to minimize older adults’ time spent engaged in sedentary behaviors.

## Figures and Tables

**Table 1:** Items of international physical activity questionnaire environmental module.

Scale Composition	Items
Access to shops	Many shops, stores, markets or other places to buy things I need are within easy walking distance of my home.
Access to public transport	It is within a 10–15 min walk to a transit stop from my home.
Presence of sidewalks	There are sidewalks on most of the streets in my neighborhood.
Presence of bike lanes	There are facilities to bicycle in or near my neighborhood.
Access to recreational facilities	My neighborhood has several free or low cost recreation facilities.
Crime safety at night	The crime rate in my neighborhood makes it unsafe to go on walks at night.
Traffic safety	There is so much traffic on the streets that it makes it difficult or unpleasant to walk in my neighborhood.
Social environment	I see many people being physically active in my neighborhood.
Aesthetics	There are many interesting things to look at while walking in my neighborhood.
Residential density	What is the main type of housing in your neighborhood?

**Table 2:** Characteristics of participants.

Variable	Category	Total Sample	
		*N* = 980 (%)	
Age	65–74	616	(62.9)
	≥75	364	(37.1)
Gender	Women	469	(47.9)
	Men	511	(52.1)
Education level	College degree or more	228	(23.3)
	Up to high school	752	(76.7)
Marital status	Married	746	(76.1)
	Unmarried	234	(23.9)
Living status	With family	851	(86.9)
	Alone	129	(13.1)
Job status	Employment	192	(19.6)
	Non-employed	788	(80.4)
Residential area	Metropolitan	504	(51.4)
	Non-metropolitan	476	(48.6)
BMI (kg/m^2^) ^a^	Non-overweight (<24)	574	(58.6)
	Overweight (≥24)	406	(41.4)
LTPA (min/week) ^b^	Sufficient (≥150)	581	(59.3)
	Insufficient (<150)	399	(40.7)
Household motor vehicles	None	184	(18.8)
	One or more	796	(81.2)
TV viewing (h/day)	<2	417	(42.6)
	≥2	563	(57.4)

Notes: ^a^ BMI = body mass index; ^b^ LTPA = leisure time physical activity.

**Table 3:** Descriptive Statistics of Perceived Neighborhood and Home Environmental Variables.

Variable	Category	Total Sample	
		*N* = 980 (%)	
**Perceived neighborhood environmental factors**			
Residential density	Low	61	(6.2)
	High	919	(93.8)
Access to shops	Good	818	(83.5)
	Poor	162	(16.5)
Access to public transport	Good	794	(81.0)
	Poor	186	(19.0)
Presence of sidewalks	Yes	608	(62.0)
	No	372	(38.0)
Presence of bike lanes	Yes	572	(58.4)
	No	408	(41.6)
Access to recreational facilities	Good	800	(81.6)
	Poor	180	(18.4)
Crime safety	Safe	149	(15.2)
	Not safe	831	(84.8)
Traffic safety	Safe	270	(27.6)
	Not safe	710	(72.4)
Social environment	Good	750	(76.5)
	Poor	230	(23.5)
Aesthetics	Good	607	(61.9)
	Poor	373	(38.1)
**Home environmental factors**			
Numbers of TV sets in home	0–1	750	(76.5)
	2 or more	230	(23.5)
TV set in bedroom	No	593	(60.5)
	Yes	387	(39.5)

**Table 4:** Odds ratios for perceived neighborhood and home environmental variables and likelihood of excessive TV viewing time.

Variables	TV Viewing ≥2 h/day		
	OR	95%CI	*p*-Values
**Perceived neighborhood environmental factors**			
**Residential density**			
Low	1.00 (ref.)		
High	0.68	0.39–1.19	0.18
**Access to shops**			
Good	1.00 (ref.)		
Poor	0.75	0.52–1.09	0.13
**Access to public transport**			
Good	1.00 (ref.)		
Poor	0.79	0.55–1.14	0.21
**Presence of sidewalks**			
Yes	1.00 (ref.)		
No	0.88	0.65-1.17	0.37
**Presence of bike lanes**			
Yes	1.00 (ref.)		
No	0.86	0.66–1.12	0.26
**Access to recreational facilities**			
Good	1.00 (ref.)		
Poor	0.85	0.60–1.20	0.36
**Crime safety**			
Safe	1.00 (ref.)		
Not safe	0.97	0.68–1.40	0.87
**Traffic safety**			
Safe	1.00 (ref.)		
Not safe	1.36	1.02–1.82	0.04 *
**Social environment**			
Good	1.00 (ref.)		
Poor	1.06	0.77–1.45	0.72
**Aesthetics**			
Good	1.00 (ref.)		
Poor	1.26	0.96-1.66	0.08
**Home environmental factors**			
**Numbers of TV sets in home**			
0–1	1.00 (ref.)		
2 or more	1.77	1.28–2.44	<0.001 *
**TV set in bedroom**			
No	1.00 (ref.)		
Yes	1.55	1.18–2.03	0.001 *

Adjusted for gender, age, marital status, job status, educational level, residential area, living status, LTPA, BMI status, household motor vehicles; OR, odds ratio; 95%CI, 95% confidence interval; * *p* < 0.05.
